# Catch my drift? Making sense of genomic intra-tumour heterogeneity^☆^^☆☆^

**DOI:** 10.1016/j.bbcan.2016.12.003

**Published:** 2017-04

**Authors:** Andrea Sottoriva, Chris P Barnes, Trevor A Graham

**Affiliations:** a Centre for Evolution and Cancer, The Institute of Cancer Research, London, SM2 5NG, UK; b Department of Cell and Developmental Biology, University College London, London, UK; c Evolution and Cancer Laboratory, Barts Cancer Institute, Charterhouse Sq, Queen Mary University of London, EC1M 6BQ, UK

**Keywords:** Clonal evolution of cancer, Neutral evolution, Selection, Clones, Intra-tumour heterogeneity, Next generation sequencing

## Abstract

The cancer genome is shaped by three components of the evolutionary process: mutation, selection and drift. While many studies have focused on the first two components, the role of drift in cancer evolution has received little attention. Drift occurs when all individuals in the population have the same likelihood of producing surviving offspring, and so by definition a drifting population is one that is evolving neutrally. Here we focus on how neutral evolution is manifested in the cancer genome. We discuss how neutral passenger mutations provide a magnifying glass that reveals the evolutionary dynamics underpinning cancer development, and outline how statistical inference can be used to quantify these dynamics from sequencing data. We argue that only after we understand the impact of neutral drift on the genome can we begin to make full sense of clonal selection.

This article is part of a Special Issue entitled: Evolutionary principles - heterogeneity in cancer? Edited by Dr. Robert A. Gatenby.

## 1 The power of free riders

Cancer development is an evolutionary process whereby the expansion of a clone can be caused by the acquisition of a new ‘driver’ mutation that causes the mutant cells to have an advantageous phenotype (e.g. increased survival) in their current microenvironment [1], [2]. But for every new driver mutation, there are many more ‘passenger’ mutations that have no effect on a cell's phenotype [3]. A clone that is ‘driven’ to higher relative frequency in the population by the driver mutation also ‘drags along’ all the passenger mutations that it has previously acquired. A commuter train provides a convenient metaphor here; much like a selected cell lineage in a cancer, a commuter train has many passengers and only one driver. This makes sense in terms of the apparent scarcity of driver mutations in cancer [4], as it takes a lot of cell divisions and a lot of random DNA mutations that come with them (i.e. the formation of a lot of passengers), before a specific tumour suppressor gene or oncogene is ‘hit’ (i.e. until a driver mutation occurs). We might think passenger mutations are the ‘baggage’ of somatic evolution: therefore since passengers do not influence the course of events in tumour evolution, why do they matter? Surely they are just ‘noise’?

That passengers are irrelevant to cancer evolution is a big misconception. Passengers are ‘free riders’ that hitchhike in a genome that carries a driver mutation, following the driver wherever it goes. But by following the passengers, in turn we can follow the drivers too: returning to the commuter train metaphor, the GPS positions of the phones of passengers in a train shows us where the train is going. On our metaphoric train, the advantage of looking at the passengers is that they are overwhelmingly more abundant than the drivers, and so their GPS signals are much easier to detect. In cancer it is challenging to determine if a mutation is a driver or not, and needs both bioinformatics approaches to determine the statistical distribution of the putative driver mutation across samples, and functional assays to demonstrate that indeed the mutation changes the phenotype of cancer cells. On the contrary, finding passengers is much easier because the genome is huge and, even for the coding part, is mostly not utilised by any given somatic cell (i.e. many mutations are synonymous, non-coding, or in a gene that is not expressed). Exploiting genetic hitchhiking to understand how a population changes over time is a very old concept, first coined by Maynard Smith in 1974 [5] and officially formalised by Gillespie in 2000 [6]. Hence, passengers are not just the ‘noise’ of evolution, but they are a rich source of information on how a population has changed over time. Passengers are signal, and the signal is loud!

## 2 What's a clone?

Tumours are thought to be ‘clonal’ because they start from a single cell. However, evolution does not stop when the tumour is initiated, but continues throughout the life-history of the malignancy. Indeed, extensive intra-tumour heterogeneity has been demonstrated across different tumour types [7], [8], which corroborates the tumour evolution paradigm [2], [9]. In cancer, the concept of ‘clone’ has been used to describe the different sub-populations of cells present in the same malignancy. This is a very useful concept that is paralleled by the concept of ‘sub-species’ in evolutionary biology. However, there's a catch. In evolutionary biology, species are defined by their phenotype, which is is usually relatively easy to measure. For example, the anatomy of extinct species is revealed by the bone structure of fossils, or other phenotypic characteristics such as body shape of extant animals, are measurable. Importantly, most of these characteristics – relatively large phenotypic differences – represent heritable traits that get passed to the next generation, so the observable differences in phenotype are largely matched by underlying differences in genotype, which we know is the basic mechanism of inheritance.

However, *molecular evolution*, the study of the change of the genome in time under evolutionary pressures [10], teaches us that phenotypes and genotypes do not always change together, and their relationship is often complex and counterintuitive. In cancer genomics, we measure genotypes but not directly phenotypes, which makes the interpretation of cancer genomic data somewhat challenging. For example, although the concept of clone is very useful, it is problematic to define formally. So what's a clone? Here are some possible definitions:

1. A group of cells that share the same (driver) mutation.
2. A group of cells with the same genome.
3. A group of cells that share the same common ancestor.
4. A group of cells that have the same phenotype (as far as we can measure).

Clearly, the first definition is somewhat ambiguous, as depending on what mutation we pick, we will identify different groups of cells. In particular, the presence of nested clones makes this definition hard to work with. The second definition is also problematic as the mutation rate is sufficiently high [11] such that we should expect a few somatic mutations to occur with each cell division, and hence that each cell should be genetically unique, so every cell in the tumour would be its own clone according to this definition. The third definition is really bad, as any two cells in a cancer share a common ancestor in the founder cell of the tumour, and in fact any two cells on earth also share a common ancestor! The fourth is a bit more useful as it is a functional definition of a clone. However, as Swanton and colleagues have shown, convergent evolution is not uncommon in cancer, and hence two subpopulations may have the same phenotype, without being closely related [12], much like the way that fish and whales have the same body shape, but they are not close relatives. Importantly however, definition #3 is usually considered the ‘standard’ in cancer biology.

A possibly more useful, yet far from perfect, definition of a clone is “a group of cells with the same phenotype, which have expressed that phenotype consistently since their most recent common ancestor”. This handles the problem of convergent evolution, and is a convenient working definition, though in most cases we note it is likely impractical to formally demonstrate temporal invariance in phenotype. Indeed even functional definitions of a clone are confounded by the continuous and often plastic (changeable) nature of phenotypes [13], [14]. Take cell division rate as an example: it can have a continuum of values (short to long) and may change in response to microenvironmental stimuli (e.g. the availability of nutrients) and such changes in rate are reasonable to expect in absence of underlying genetic change. Thus, definition #3, when the common ancestor in question is specified, at least provides a less ambiguous definition of a clone.

## 3 What happens when nothing happens?

**Fig. 1:**
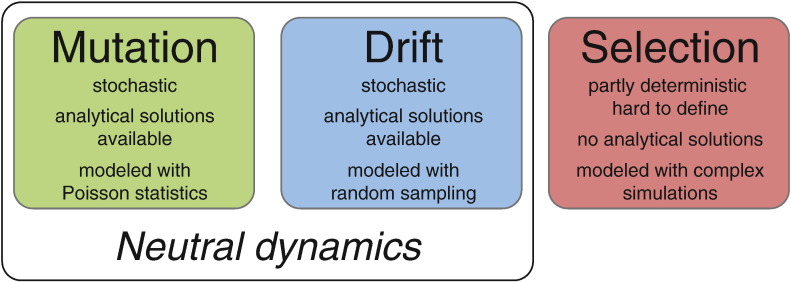
The dynamics of somatic evolution. Somatic evolution is the result of the interplay of three fundamental forces: random mutation, random drift, non-random selection. Random mutations are inherently stochastic, but can be handled with existing mathematical tools such as Poisson statistics. Drift is also stochastic, and can be modelled with random sampling. Selection instead is non-random, but comprehensive mathematical tools to describe the result of selection are still lacking. When selection is not in operation, only the first two processes act, and the combination of random mutation and random drift together are what is defined as neutral evolution.

Somatic evolution results from the interplay of three fundamental forces: random mutations, random drift and non-random selection (Fig. 1). For sake of simplicity, throughout this document we will refer to selection as positive (Darwinian) selection, which triggers the outgrowth of new clones. Mutations provide the substrate for genetic and phenotypic variation, hence this process *increases* heterogeneity. Drift and selection change the frequency of alleles (and of clones) in a population, making some larger or even dominant, and others to go extinct. Drift does this at random, while selection does it based on reproductive fitness. Both processes generally *reduce* heterogeneity.

Drift results from one lineage randomly having more offspring than another (perhaps due to random cell killing) and intuitively drift has a significant effect on allele frequencies in small populations but a proportionally much smaller effect in large populations. For example, if a tumour is composed of only four cells, which all divide and then half their offspring are killed at random (so only 4 cells remain), then it would not be unlikely to find that the surviving four cells came from just two of the ancestors. Selection occurs when individuals show different proliferation or survival rates: they show ‘functional variation’. If there is no difference in proliferation or survival, a population is defined as functionally ‘homogeneous’: all individuals have equal *fitness* in their current context.

In a homogeneous population, we may confidently state that there is only one ‘clone’. But, while the phenotype is ‘stable’ in a homogenous population, what happens to the genotype? In other words, what is happening while apparently nothing (no phenotypic change) is happening? In the absence of clonal selection, genotypes cannot ‘stay still’. In such a scenario, the two forces of random mutation and drift will still be at play, respectively introducing new variants into the population and altering their frequency. Hence the genomic variation within the population will increase as time passes. While the population could be considered a single ‘clone’ from a functional point of view (definition #4 above), it is actually constituted by a multitude of different lineages, each with its own unique set of genomic mutations. In this case, the evolutionary dynamics at play are defined as *neutral* (Fig. 1) as no lineage behaves differently than another. Importantly, the ‘dark side’ of this process is that when the environment changes, neutral variation may become functional in the new microenvironment, and so potently induce selection of the lineage. In fact, the concept that variation is pre-existing and ‘neutral’ in origin is the very essence of Darwinian evolution, and was formally demonstrated experimentally for the first time by Luria and Delbruck with their famous and exquisitely elegant experiment in 1943, showing pre-existing resistance in bacterial populations [15], for which they won the Nobel prize.

Paradoxically, out of the three fundamental processes in evolution, although selection seems to be the easiest to understand, it actually produces the most complex patterns. Moreover, whereas we have the quantitative mathematical tools to understand random mutations (e.g. Poisson statistics) and genetic drift (e.g. Markov processes), a general mathematical formalism for selection is still to be defined. Although extensive work has been done to determine timing of clonal sweeps and accumulation of selected variants [16], [17], solutions for the allele frequency distributions within a population under selection are generally an unsolved problem in population genetics. This is in part due to the fact that we do not know what the genotype-phenotype map is in cancer, and it remains unclear to what extent we *can* ever know it, considering the potentially limitless combinations of genotypes and environments.

**Fig. 2:**
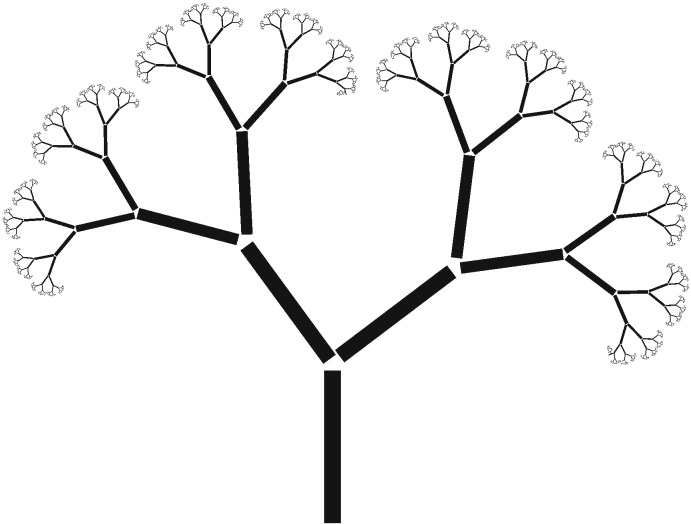
The fractal pattern of neutral evolution. In the absence of selection, genotypes are free to mutate as the tumour grows, generating a well-defined fractal pattern in the phylogenetic history of the malignancy, with more and more rare mutations (rare branches) at lower and lower frequencies. This pattern is characterised by a 1/f distribution of the allele frequencies of mutations within a population.

Importantly however, the change in allele frequencies in a neutrally evolving population is analytically tractable [15], [18], even when the population is exponentially growing (such as cancer) [19], [20], [21]. The frequency distribution of mutations in an exponentially growing and neutrally evolving population has the solution:

$$M\left(f\right)\sim \frac{1}{f}$$

where *f* is the frequency of a mutation within the population and *M(f)* is the cumulative number of mutations. This dynamic behaviour produces a fractal-like structure in the phylogeny of the population, where the phylogenetic tree doubles its numbers of branches each time the cell population doubles (Fig. 2). This is because in a neutrally evolving population there is no selection acting that would act to ‘prune’ the branches of the tree. So when apparently ‘nothing happens’ to the phenotype, actually a lot of things happen to the genotype. In fact, under this scenario, referred to as ‘neutral evolution’, the maximal genetic variation is created, as there's no selective force removing variation. Consequently, neutrally evolving tumours may be the most ‘evolvable’, as they are likely to generate pre-existing variation that could be adaptive if the environment were to change. Neutral evolution is considered the null model of molecular evolution against which the effects of selection can be distinguished [22], [23], [24].

**Fig. 3:**
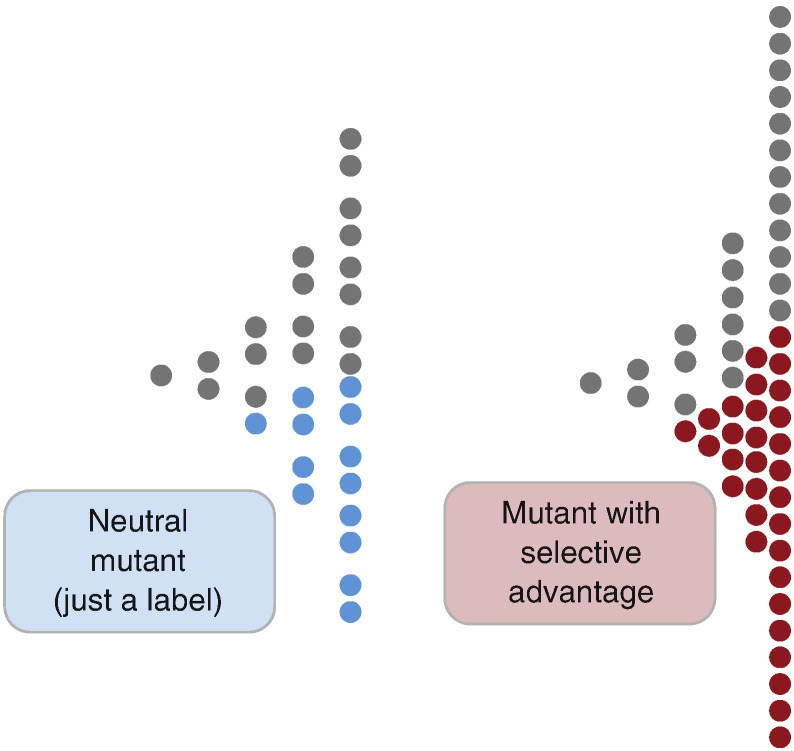
Neutral evolution versus selection. When neutral dynamic are operating, new mutations in the genome represent just labels for individual cell lineages and so the frequency of new mutations decreases at a rate inversely to tumour size (this 1/f pattern of allele frequencies is characteristic of neutral growth). In the case of selection instead, both subclonal driver and passenger mutations are carried at higher frequency than expected under neutrality, generating signatures of clonal outgrowth (‘too many’ mutations at high frequency) that distinguishes the pattern of allele frequencies under selection from the neutral case. Generation time goes from left to right, starting from a single cell that expands exponentially.

Under neutral evolution, all mutations are just a ‘label’ for different cell lineages, whereas under selective evolution, the frequency of the neutral labels are changed by the outgrowth of new clones, with selection increasing the frequency of both driver and passenger alterations in a given population (Fig. 3). If selection is operating, the distribution of allele frequencies in a population will not fit the null model, and so fitting data to the null model provides a convenient test for the presence of on-going clonal selection in a population.

## 4 Measuring evolution

Many sophisticated bioinformatics methods have been developed in recent years to analyse the wealth of data from cancer genomic profiling [3]. Those techniques have shed new light on the complexity of the cancer genome [25], and provided unprecedented insight on the genomic alterations that initiate and drive malignancies [4]. Similar statistical methods have also been applied to study the subclonal evolution of tumours, revealing intricate clonal architectures [26], [27], [28]. However, although extremely useful, these approaches have limited power for understanding how the tumour changes *over time*. That is because, although statistically rigorous, these methods are not mechanistic: they do not model the evolutionary dynamics themselves, but rather provide a snapshot of the current clonal composition of the tumour.

Key questions in cancer evolution are: How did the tumour grow in a specific patient? How did one cell become 100 billion cells? What patterns do we expect to see in the data depending on the different evolutionary trajectories of a tumour? Unsurprisingly, those patterns are often complex and counterintuitive, and so mathematically-described mechanistic models based on evolutionary theory provide a valuable complement to statistical bioinformatics approaches when interpreting cancer genome data [29], [30]. Understanding tumour evolution with mechanistic models is important because it allows assumptions about the underlying evolutionary dynamics to be formally stated, thus allowing an evolutionary hypothesis to be statistically tested. Moreover, mechanistic models permit us to *measure* new parameters of tumour evolution, particularly those parameters that describe evolution *over time*. This is particularly important in the case of human malignancies, for which longitudinal measurements are difficult, and sampling is limited by ethical and technical issues.

For example, in the case of neutral growth, the complete analytical solution for variant allele frequency distributions in the case of one cell becoming billions of cells is the following:

$$M\left(f\right)=\frac{\mu }{\beta }\left(\frac{1}{f}-\pi \right)$$

where *π* is the ploidy of the tumour (number of copies of the genome per cell), *μ* is the mutation rate per division and *β* is the ‘lineage survival’ rate per division, accounting for cell death and turnover. Hence, the slope of the line in this linear equation is precisely the ‘effective mutation rate’ *μ*/*β* – in other words the rate of accumulation of mutations for each new cell lineage generated. This means that with this model we can not only determine if a population is growing neutrally or not, but in the case it is indeed evolving neutrally, we can measure its mutation rate *in vivo* [21].

Multi-region sampling of a tumour allows more detailed characterisation of the evolutionary dynamics. The tumour subclonal architecture could be very complex and distinct regions of the neoplasm may be characterised by radically distinct dynamics. Multi-region sequencing allows one to reduce sampling bias of single biopsies and unravel the tumour evolutionary complexity on another scale [7]. With multi-region profiling, both the allele frequency distribution of mutations (both passengers and drivers) and the physical distribution of those mutations around the tumour can be analysed. Early mutations will be common in a tumour, whereas later mutations will be more spatially isolated – this kind of ‘mutational ordering’ analysis provides rich insight into the pattern of mutation and clonal expansion that have shaped a tumour's clonal composition [12], [31], [32], [33], [34], [35], [36], [37], [38], [39], [40], [41].

However, as complexity increases – such as when considering time-varying changes in the selective microenvironment – simple and elegant analytical solutions do not exist, and so to describe tumour evolution we are forced to turn to stochastic simulations where in principle arbitrarily complexity can be implemented [29], [38], [42], [43], [44], [45], [46], [47]. But how do we fit complex stochastic models to complex multi-dimensional data?

The most appropriate technique in this case is Bayesian inference – a technique that allows us to ask how well a particular model describes the available data, given our prior knowledge about the parameter values.

In particular, many major Bayesian inference techniques, such as Markov Chain Monte Carlo (MCMC) [48], rely on the calculation of a model's *likelihood* (the probability that a model will produce the observed data) to estimate posterior parameter distributions (the parameter values that best describe the available data). For complex models it is often not possible to compute the likelihood because the model is too complex and/or non-analytical in essence (e.g. Agent based models that describe each cell independently [42] that cannot be expressed in terms of a simple likelihood equation). To overcome this ‘non-computability’ problem, a powerful and elegant likelihood-free technique that has gained popularity amongst population geneticists in recent years is Approximate Bayesian Computation (ABC) [49], [50], [51], [52]. Because ABC does not require the computation of likelihoods, it can be applied to models of arbitrary complexity.

The idea behind ABC is to make a ‘guess’ (based on prior beliefs and/or knowledge) of the correct values a model's parameters, and then simulate the model with these guessed parameters and evaluate how well the model's output recapitulates the experimental data at hand. If the guessed parameters are ‘good’ (e.g. they produce model output faithful to the experimental data) then the guess is added to a list of acceptable parameters. If the guess is ‘bad’ (produces unrealistic model output) then it is discarded. This process of (1) guessing parameters, (2) simulating the model with the guessed parameter values, and (3) accepting/rejecting the guess on the basis of how well the data is recapitulated, is repeated many times (typically millions to billions of trials) until a comprehensive list of acceptable parameters is drawn up. This list of acceptable parameters is called the ‘posterior distribution’, and by looking at the range of values within the posterior distribution we can assess which parameters best represent the data.

The remainder of this section presents ABC in more formal mathematical terms. Readers who are not interested in the technical details can skip to the next section where we explain an application of ABC to cancer data.

ABC statistical inference methods came from earlier similar approaches based on ‘rejection algorithms’ [53], [54]. In ABC the computation of the likelihood is substituted by a rejection step where some distance function ρ is used to evaluate the ‘closeness’ of the model's output to the available data, according to the following scheme:

1. Sample the parameters *θ* from the prior distributions *P*(*θ*)
2. Simulate virtual data *D*′ from the stochastic model *M* with the parameter input *θ*
3. If *ρ*(*D*, *D*^′^) < *ϵ*, accept *θ*
4. Go to (1)

The rejection step 3 avoids computing the likelihood by accepting parameters from the prior only if they generate virtual data that are similar to the observed data, given a certain distance measure *ρ* and tolerance *ϵ*. Collecting the accepted *θ* is equivalent to sampling from the posterior distribution *P*(*θ* | *ρ*(*D*, *D*^′^) < *ϵ*). The problem with this algorithm is that for complex multi-dimensional data, the acceptance step 3 may have an extremely low acceptance rate, which means that a very large number of simulations of the model must be performed to well approximate the posterior distribution. In these cases it is often necessary to use summary statistics *S*(*D*) of the data rather than the data themselves and calculate the distance as *ρ*(*S*(*D*), *S*(*D*^′^)) accordingly. What we obtain is an approximated version of the posterior distribution that corresponds to *P*(*θ* | *ρ*(*S*(*D*), *S*(*D*^′^)) < *ϵ*). It is mathematically proven that for *S*(∙) sufficient and *ϵ* → 0, such approximation converges to the exact posterior [50]. The sufficiency of the summary statistics given a model and a parameter *θ* means that the statistic contains the maximal amount of information about the data (in that *P*(*D* | *S* , *θ*) is independent of *θ*). Unfortunately, sufficient statistics rarely exist for realistic situations such as non-exponential family or agent-based models, hence one must rely on a combination of multiple summary statistics that (hopefully) provides a good approximation of the posterior. The magnitude of the approximation introduced by the method depends on *S*(∙), *ρ*(∙) and in particular ε that has to be chosen as a trade-off between accuracy and computability. Given the small *ϵ*, to generate a good posterior we need to draw a very large number of simulations because of their small chance of being accepted. This is the chief bottleneck of the method that requires simulating an instance of the model extremely quickly. For this reason, in this type of approach the computational performance of the model is crucial and determines the precision with which we can derive the posterior distribution. Despite the approximation, ABC is extremely useful and permits us to perform inference in many cases where traditional statistical methods simply cannot be used, including complex agent-based models of cancer evolution.

## 5 The ABC of colon cancer

We applied this combination of genomic data and statistical inference to study the evolution of colorectal cancer. In order to deconvolute the subclonal architecture of colorectal cancer at the single-clone resolution, in a recent study we performed genomic profiling on 349 individual colorectal *glands* (small tubular structures derived from a small number of stem cells) from 15 colorectal tumours [38]. Our computational modelling showed that the patterns of genetic heterogeneity within the tumours were consistent with a ‘Big Bang’ expansion in which the tumour grew as a single expansion, populated by a large number of early-arising clones that were coexisting for long periods of time due to the lack of stringent selection. A stepwise accumulation of driver mutations could not explain the data, instead the subclonal dynamics could be governed by weak selection that was insufficient to drive large clonal expansions over short times. Together, this implied that the majority of *observable* intra-tumour heterogeneity was generated early in the primordial tumour, long before the tumour reached a clinically detectable size. The lack of stringent selection meant that newly generated mutations in an already established tumour effectively only experienced drift, and so were unlikely to reach a detectable size in the tumour.

**Fig. 4:**
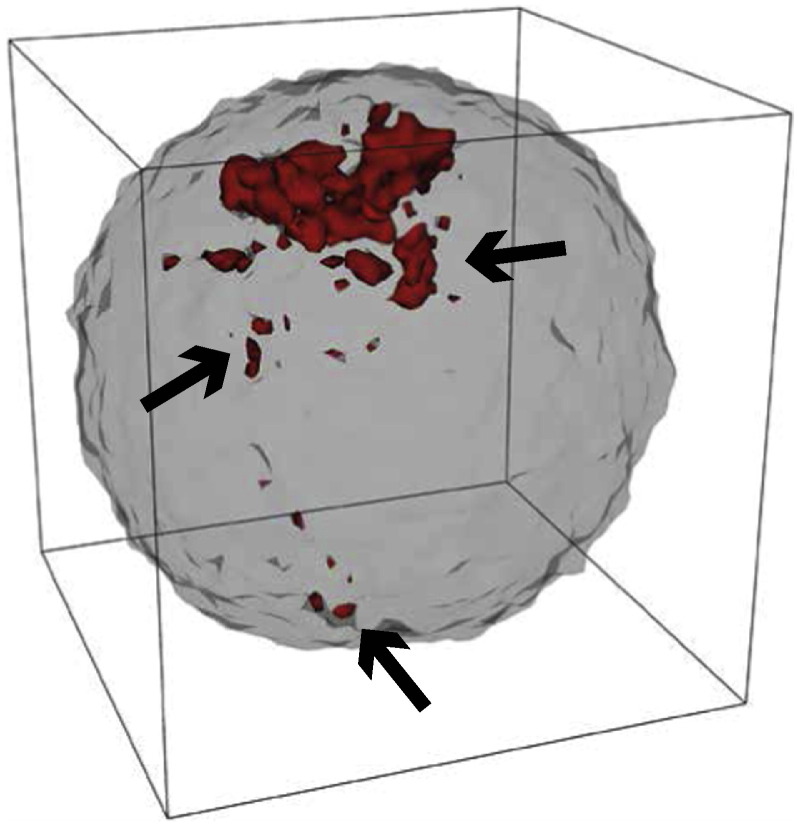
Spatial properties of growing clones. When tumours grow in a disordered fashion, when cell push each other around by means of proliferation pressure, characteristic patterns of subclonal intermixing are spontaneously generated. In this simulated case, a new mutation in red originated early during the growth of the tumour, but was scattered by the disordered growth dynamics, and propagated to far away locations in the malignancy by the growth of the neoplasm. This occurs just by means of disordered growth, with no active migration of cells and it is an indication of a potentially invasive phenotype.

Importantly, spatial profiling also allowed to discover that carcinomas, which are malignant lesions, where characterised by clonal intermixing in distant parts of the tumour, whereas adenomas, which are instead benign lesions, were not. Computational modelling showed that this intermixing was likely a consequence of abnormal cell mobility in the early cancer (Fig. 4). The Big Bang model has also been observed in breast cancer [37], and hepatocellular carcinoma [34], and clonal intermixing has also been observed subsequently in breast cancer [35].

Importantly, the use of ABC allowed us to make inferences about the *phenotypes* of colon cancer cells (e.g. selection for particular lineages) using only the (quantitative) information about clone size and location provided by the genomic data.

## 6 Conclusion

The cancer genome is shaped by three fundamental components of evolution: mutation that generates new variation in the population, and drift and selection that underlie the expansion and contraction of the clones. While clonal selection is arguably the most important of these processes – not least because the selection of mutants capable of migrating and growing in distant regions of the body underpins deadly metastasis – it is unfortunately also the most challenging of the processes to mathematically define and quantify. Moreover, because drift can also lead to clones expanding within a tumour, we must be careful to discern the effects of drift and selection. The appropriate combination of bioinformatics approaches coupled with mechanistic mathematical modelling of the underlying evolutionary processes (encompassing all three of mutation, drift and selection) provides a tractable way to make sense to the wealth of data encoded in the cancer genome. And because the cancer genome is shaped by the evolutionary dynamics of tumour clones, the evolution of cancer cell *phenotypes* can begin to be understood by using statistical inference to parameterise mechanistic models of cell behaviours against the genomic data. It is a cell's phenotype, not its genotype, that is the ultimate driver of cancer evolution, but irrespective of the nature of the driver itself, the history of cancer evolution is inevitably written in the genome. With the right tools, genomic measurement therefore provides a surreptitious handle with which we can understand phenotype evolution.

## Statement of author contributions

AS, CB and TG co-wrote the manuscript.

## Transparency document

Transparency document. Image 1
